# Australian Youth Self-Harm Atlas: spatial modelling and mapping of self-harm prevalence and related risk and protective factors to inform youth suicide prevention strategies

**DOI:** 10.1017/S2045796024000301

**Published:** 2024-09-09

**Authors:** E. Hielscher, K. Hay, I. Chang, M. McGrath, K. Poulton, E. Giebels, J. Blake, P. J. Batterham, J. G. Scott, D. Lawrence

**Affiliations:** 1 QIMR Berghofer Medical Research Institute, Herston, QLD, Australia; 2 School of Public Health, Faculty of Medicine, The University of Queensland, Brisbane, QLD, Australia; 3 Flourish Australia, Sydney Olympic Park, NSW, Australia; 4 School of Psychology and Counselling, Faculty of Health, Queensland University of Technology, Brisbane, QLD, Australia; 5 Roses in the Ocean, Brisbane, Australia; 6 Metro North Mental Health, Royal Brisbane and Women’s Hospital, Herston, QLD, Australia; 7 Centre for Mental Health Research, The Australian National University, Canberra, ACT, Australia; 8 Child Health Research Centre, The University of Queensland, Brisbane, QLD, Australia; 9 Child and Youth Mental Health Service, Children’s Health Queensland, Brisbane, QLD, Australia; 10 School of Population and Global Health, The University of Western Australia, Perth, WA, Australia; 11 School of Population Health, Curtin University, Perth, WA, Australia

**Keywords:** adolescence, geospatial, prevalence, self-harm, spatial analysis, suicide

## Abstract

**Aims:**

Suicide prevention strategies have shifted in many countries, from a national approach to one that is regionally tailored and responsive to local community needs. Previous Australian studies support this approach. However, most studies have focused on suicide deaths which may not fully capture a complete understanding of prevention needs, and few have focused on the priority population of youth. This was the first nationwide study to examine regional variability of self-harm prevalence and related factors in Australian young people.

**Methods:**

A random sample of Australian adolescents (12–17-year-olds) were recruited as part of the Young Minds Matter (YMM) survey. Participants completed self-report questions on self-harm (i.e., non-suicidal self-harm and suicide attempts) in the previous 12 months. Using mixed effects regressions, an area-level model was built with YMM and Census data to produce out-of-sample small area predictions for self-harm prevalence. Spatial unit of analysis was Statistical Area Level 1 (average population 400 people), and all prevalence estimates were updated to 2019.

**Results:**

Across Australia, there was large variability in youth self-harm prevalence estimates. Northern Territory, Western Australia, and South Australia had the highest estimated state prevalence. Psychological distress and depression were factors which best predicted self-harm at an individual level. At an area-level, the strongest predictor was a high percentage of single unemployed parents, while being in an area where ≥30% of parents were born overseas was associated with reduced odds of self-harm.

**Conclusions:**

This study identified characteristics of regions with lower and higher youth self-harm risk. These findings should assist governments and communities with developing and implementing regionally appropriate youth suicide prevention interventions and initiatives.

## Introduction

Suicide prevention strategies in Australia and worldwide have shifted in recent years, from a national approach to one that is tailored to regional needs. The need to understand suicidal behaviour within small-area geographies is supported by a growing spatial epidemiology literature. However, most of these geospatial studies have focused on suicide deaths, which may not fully capture the data required for a comprehensive understanding of suicide prevention needs (Rosychuk *et al.*, 2016; Torok *et al.*, 2019). Targeting non-suicidal self-harm and suicide attempts could substantially contribute to reductions in suicide rates (Hawton *et al.*, 2020; Townsend, 2019), given that people who present to hospital for self-harm are up to 30-times more likely to die by suicide than the general population (Cooper *et al.*, 2005; Hawton *et al.*, 2020). Also, few studies have used national data focused on the priority population of young people, who report the highest rates of self-harm (Robinson *et al.*, 2016a, 2016b). It is therefore timely to investigate regional variation in self-harm prevalence (non-suicidal and suicidal) and related factors in Australian youth, thereby identifying localised priority targets and regions of need.

Previous studies show substantial differences between regions, in terms of suicide incidence and related factors. In the Australian context, national data show highest suicide rates in the Northern Territory for both youth (<25 years) and adults (30.3 and 22.5 per 100,000 persons per year respectively), followed by Western Australia (11.2 and 20.2) and Queensland (10.0 and 19.0) (Robinson *et al.*, 2016b). The few non-fatal self-harm Australian studies also show substantial regional variation (Inder *et al.*, 2014; Too *et al.*, 2019, 2017; Torok *et al.*, 2017), with metro-rural gradients (of increasing prevalence) for suicide attempts (Too *et al.*, 2017) but not for self-harm (irrespective of intent) (Torok *et al.*, 2017). These self-harm studies have largely been conducted in state-specific (New South Wales or Western Australia) adult samples, and nearly all have used routinely collected hospital data. As self-harm episodes typically do not result in a hospital admission (Hawton *et al.*, 2012), hospital data underestimate overall self-harm rates by up to 60% (Clements *et al.*, 2016). Also, no studies have reported geographic variation of non-suicidal and suicidal self-harm separately, potentially obscuring results (Torok *et al.*, 2017). These gaps and trends are also seen in the limited international literature, where most studies report on spatial patterns of self-harm hospitalisations in relatively localised areas (Congdon, 2012; Corcoran *et al.*, 2007; Lin *et al.*, 2019; Rosychuk *et al.*, 2016).

In addition to geographic variation in prevalence and incidence, previous studies show evidence of areas of spatial clustering of self-harm and suicide. These areas represent the most likely candidate regions for intensive prevention and intervention efforts. Using Australia data, Hill *et al.* (2020) found eight spatial-temporal clusters of high relative risk of youth suicides (10–24 years) in New South Wales, Northern Territory, Queensland, Western Australia, Tasmania, and Victoria. Similarly, Robinson et al. (2016b)identified five spatial youth suicide clusters across most Australian states and territories. Three of the five youth suicide clusters were identified in remote areas (Robinson *et al.*, 2016b); consistent with other Australian (Cheung *et al.*, 2012; Cheung *et al.*, 2014; Qi *et al.*, 2010) and international studies in Canada, Taiwan and the United States (Chang *et al.*, 2011; Fontanella *et al.*, 2015; Ngui *et al.*, 2014). There is also evidence of spatial clustering of self-harm behaviours (Too *et al.*, 2019, 2017; Torok *et al.*, 2017) but none have been nationwide investigations in Australia (and only a few internationally, Congdon, 2012; Corcoran *et al.*, 2007; Gould *et al.*, 1994), and few have disaggregated suicide attempts from suicide deaths.

In terms of relevant risk and protective factors, Aboriginal and/or Torres Strait Islander Status, prior exposure to suicide, low socio-economic status, and unemployment level have all been associated with Australian regions with increased prevalence of youth suicide (Hill *et al.*, 2020; Robinson *et al.*, 2016b). Similar factors have been identified internationally for self-harm (Corcoran *et al.*, 2007; Lin *et al.*, 2019) and suicide (Chang *et al.*, 2011; Fontanella *et al.*, 2018; Hsu *et al.*, 2015), including various socio-economic and social fragmentation indices (e.g., unemployment, households privately renting, single parent households). In terms of the latter, the effects of economic deprivation and social fragmentation (on small-area self-harm rates) have importantly been shown to be modified by geographic area (in the UK and Taiwan, Chang *et al.*, 2011; Corcoran *et al.*, 2007); implying that the findings of city or regional areas may not generalise to other areas within the same country. Few spatial studies have included psychological variables, a key predictor of self-harm and suicide (Inder *et al.*, 2014; Qi *et al.*, 2012), due to data limitations at lower-level geographies. Using a broad remoteness indicator, Inder *et al.* (2014) found psychiatric disorders (at the individual level) was the main determinant of 12-month suicide attempts across all geographical regions (metropolitan and rural), indicating that psychological variables may have more of a universal effect on self-harm and suicidality. Research which examines a broad range of psycho-social and socio-economic factors related to youth self-harm, and how such relationships vary across the nation, would help identify priority prevention targets in distinct geographic regions.

### Aims

This study aimed to produce the first nationwide estimates of regional variability of self-harm (non-suicidal and suicidal), and identify related risk and protective factors, in Australian adolescents. This was achieved by analysing a nationally representative youth survey (Young Minds Matter [YMM]) and Census data to produce small area self-harm prevalence estimates, and to examine small area relationships with socio-demographic and psycho-social factors. In doing so, this study intended to identify characteristics of regions with lower and higher youth self-harm risk. We hypothesised there would be (1) regional variation in the prevalence of youth self-harm and (2) small-area clustering in the predicted prevalence, across the nation. We also hypothesised that (3) the distribution and impact of social determinants (e.g., socio-economic, housing condition) would differ by remoteness, whereas the impact of psychological factors would not differ by remoteness.

## Methods

### Data

Data used in this study were from the YMM survey (Hafekost *et al.*, 2016; Lawrence *et al.*, 2016), or the Second Australian Child and Adolescent Survey of Mental Health and Wellbeing, a nationally representative random sample of Australian households with children (aged 4–10 years) and/or adolescents (aged 11–17 years), with data collected by the Telethon Kids Institute between 31 May 2013 and 10 April 2014. The design, sampling and survey interview methods are described extensively elsewhere (Hafekost *et al.*, 2016; Lawrence *et al.*, 2016). Briefly, the survey employed area-based random sampling with voluntary recruitment and consent of households in scope where there was at least one child aged 4–17 years (up to but not including the age of 18) who were residing in private dwellings in Australia. The overall response rate to the survey was 55%, with 6,310 families with children aged 4–17 years participating. In addition, 2,967 (89%) of the consenting young people aged 11–17 years completed a self-report questionnaire, with 2,655 12–17 years olds completing questions about self-harm and suicidal behaviour (Zubrick *et al.*, 2016). Comparisons with Census data show that the YMM sample was broadly representative of the overall Australian population, except for age of the child (higher proportion of 4–7-year-olds) and number of children in the household (Lawrence *et al.*, 2016).

### Measures

This study focused on ‘non-suicidal self-harm’ and ‘suicidal ideation/plans/attempts’ among Australian adolescents (12–17 years), both of which were captured via self-report in the YMM nationwide survey. The YMM self-report survey questions probed lifetime and 12-month occurrence of (1) non-suicidal self-harm (‘Have you deliberately done something to cause harm or injury, without intending to end your own life during the past 12 months?’) and (2) suicidal ideation, plans and attempts (‘Did you attempt suicide during the past 12 months?’). The current study’s primary outcome was 12-month self-harm irrespective of intent (i.e., non-suicidal self-harm or suicide attempts in previous 12 months). Secondary 12-month outcomes were (1) non-suicidal self-harm, (2) suicide attempts, (3) suicidality (ideation, plans or attempts), and (4) suicidal ideation/plans only (suicide attempts excluded from this variable).

Potential risk and protective factors were identified a priori, based on a review of relevant literature (Cantor and Neulinger, 2000; Hawton *et al.*, 2012; Hill *et al.*, 2020; Inder *et al.*, 2014; Too *et al.*, 2017; Torok *et al.*, 2017) and expert knowledge. See Table 1 (and Appendix 1) for variables used in this study from YMM, and the 2011 and 2016 Census of Population and Housing, the most comprehensive snapshot for all geographic regions of Australia (Australian Bureau of Statistics [ABS], 2011, 2016). 2011 Census data were the baseline population data at the time of the YMM survey, and its main purpose in the current study was to implement the synthetic estimation. Equivalent 2016 Census data with 2019 population counts were used to extrapolate self-harm synthetic estimates for 2019.

**Table 1.** Key risk/protective factors of interest included from the Young Minds Matter and Census data, at an individual, family, and area-level

Data source	Demographic (self-reported)	Psychological (self-reported)	Risky behaviours (self-reported)	Family information (parent-reported)	Area-level (SA1)
Young Minds Matter survey (2013−2014) *N* = 2,655	Age Sex Aboriginal and/or Torres Strait Islander Status Country of birth Highest level of education	Psychological distress (past 4 weeks): Kessler-10-item psychological distress scale (K10) Major depression (past 12 months): Diagnostic Interview Schedule for Children (DISC-IV). DISC-IV is a highly structured diagnostic interview based on DSM-IV criteria, designed to assess ≥30 psychiatric disorders in children and adolescents, and can be administered by ‘lay’ interviewers after a short training period (Shaffer et al., 2019). Self-esteem: Adolescent Self-Esteem Questionnaire Psychosis screener (past 12 months): items from DISC-IV Disordered eating Insufficient sleep (<8 hours per night)	Past month alcohol use Lifetime illicit substance use Bullied or cyber bullied (past 12 months)	Parent country of birth Parent education and employment Household income Housing tenure^a^ Family functioning McMaster Family Assessment Device Family blending Family connectedness (self-rated as always, mostly, sometimes, hardly ever/never)	Green space access and quality Housing condition School Index of Community Socio-Educational Advantage (ICSEA) Small area (SA1-level) synthetic estimates of internalising disorder prevalence (i.e., anxiety, depression). Appendix 4 Aggregated data from person-level YMM data
ABS Census (2011, 2016) *N* = ∼10 million households	–	–	–	–	Country of birth Aboriginal and/or Torres Strait Islander Status Parent country of birth Household income Parent education and employment Housing tenure SEIFA indices^b^ ARIA remoteness index (major cities, inner regional, outer regional, remote, very remote)^c^

a Housing tenure – the financial and legal arrangements of a house or other dwelling. This includes: owned outright, owned with mortgage, rental (non-government), rental (state), and rental (other).

b Socio-Economic Indexes for Areas (SEIFA) (ABS Census data; 2011, 2016). This includes: Index of Relative Socio-economic Disadvantage (IRSD), Index of Relative Socio-economic Advantage and Disadvantage (IRSAD), Index of Economic Resources (IER) and Index of Education and Occupation (IEO).

c ARIA stands for Accessibility and Remoteness Index of Australia. ARIA is widely used within the Australian community and has become recognised as a nationally consistent measure of geographic remoteness, ranging from major cities to very remote.

Note, more details about the rationale for inclusion of these variables, their description, and rationale for their cut-off scores in the current study are provided in Appendix 1.

### Modelling

The YMM survey did not collect data from every community; a sample of 540 was included (∼1% of total Statistical Area Level 1s [SA1s] across Australia). There are 57,523 SA1 regions covering the whole of Australia without gaps or overlaps, where each SA1 contains on average 400 persons (ranging from 200 to 800; Australian Bureau of Statistics [ABS], 2017). Statistical areas are aggregated to higher levels (SA2, SA3, SA4, Primary Health Network, state). Australian Primary Health Networks (PHNs) comprise 31 geographical areas which share common healthcare administration. States with larger populations have several PHNs; smaller states and territories have only a single PHN. Key modelling steps of the current study are summarised below, where all steps were conducted using Stata v.15. All modelling (including the derivation of small-area synthetic estimates) incorporated the YMM survey sampling weights and complex sampling scheme, where the survey data had a multilevel structure with households nested within SA1s, nested within SA2s, nested within SA3s, nested within PHNs within states. Outcomes of interest were modelled using generalised linear modelling, specified using binomial family and the logit link (a more detailed summary of modelling steps is provided in Appendix 1).

#### Modelling step 1: Descriptive analyses for estimated self-harm prevalence

Direct estimates of self-harm prevalence in the past 12 months (nationwide) with exact 95% confidence intervals (CIs) were presented overall, by age group and sex, and by state and stratum. Crude point-prevalence estimates (SA1 level) were used in modelling to derive synthetic estimates.

#### Modelling step 2: Predictive modelling (nationwide) to determine which factors best predict self-harm

Step 2 involved determining which person-level and area-level variables were most predictive of self-harm, and therefore should be considered candidates for the out-of-sample predictive modelling (Step 1 in the synthetic estimation subsection below). Associations with self-harm were firstly assessed in person-level models before building area-level models. Variables of interest were identified a priori (see Table 1). Logistic regression models were used to assess univariable associations. Eligible variables with *p*-values <.20 were entered and tested in a multivariable model and purposeful selection process was applied. Akaike’s information criteria was used to select the best-fitting final base model.

### Synthetic estimation

To investigate regional variation of self-harm prevalence across the nation, we used small area estimation (SAE), which utilises out-of-sample predictions to derive model-based ‘synthetic’ prevalence estimates for areas with no survey respondents (Maiti *et al.*, 2016; Rao and Molina, 2015). This is achieved by incorporating measures common to both the measured (YMM) and unmeasured population (Census data available for all regions). The number of YMM survey respondents per SA1 across Australia was small (only ∼1% of the 57,523 SA1s), and therefore, any ‘design-based’ estimate of self-harm prevalence would be highly imprecise and of limited use. Model-based estimates (produced via multi-level SAE modelling) instead borrow strength from multiple data sources and are useful to produce prevalence estimates for small geographical areas with limited data. Previous SAE studies with similar amounts of survey data (1–4% of the total population) have demonstrated precision, validity, and reliability of their SAE synthetic health indicator estimates (Viljanen *et al.*, 2022; Zhang *et al.*, 2015). Main spatial unit of the current analysis was SA1 (lowest geographic unit available) for place of usual residence. Synthetic estimation was conducted using Stata v15’s ixtmelogit command (for mixed effects regression modelling), and mapping of estimates was conducted using ArcGIS Pro. Key synthetic estimation steps outlined below (more details provided in Appendix 1).

#### Synthetic estimation step 1: Building an SA1-level (mixed effects) model and obtaining out-of-sample predictions

Self-harm prevalence estimates were obtained from the original individual-level YMM survey data, taking account of the complex sampling scheme. From the estimated point prevalence (see modelling steps in previous section) and number of 12–17-year-olds in each SA1 (from 2011 Census), a crude estimated count for the number of 12–17-year-olds with each self-harm outcome for each SA1 was derived for each SA1 with survey data. The effects of auxiliary Census variables and clustering of prevalence at different hierarchical levels was explored by fitting mixed-effects logistic regression models. For the final model, a two-level grouped logistic regression model was specified using SA1 as the unit of analysis and a random intercept for each PHN region (i.e., local health boundaries); i.e., a final model of SA1-level fixed effects and PHN-level random effects. Smoothed self-harm estimates obtained from this multilevel predictive modelling were benchmarked using direct (PHN-level) prevalence estimates derived from YMM (Appendix 1). Predicted prevalence estimates for each SA1 were updated to June 2019 using 2016 Census data and estimated resident population counts. Prevalence estimates for other geographic units (SA2, SA3, SA4, PHN, State) were aggregated from SA1 estimates.

#### Synthetic estimation step 2: Validation using external suicide dataset

To test the validity of SAE estimates, suicide death data from the National Mortality Database (available for all SA2s; 2010–2019) were compared to the current study’s SA2 self-harm prevalence estimates (Appendix 2). The National Mortality Database contains cause of death information for all deaths registered in Australia since 1964.

#### Synthetic estimation step 3: Mapping smoothed ‘synthetic’ prevalence estimates

Smoothed SAEs of self-harm prevalence (by ‘smoothed’ we mean area-level probabilities of self-harm that were shrunken towards the mean) were imported into ArcGIS Pro. Choropleth maps were created (ranging from SA2- to state-level). Density measures, i.e., cases per sq km, were mapped separately. Spatial distribution of prevalence was statistically examined nationwide using the Hot Spot Analysis (Getis-Ord Gi*) tool which pinpoints locations of statistically significant high- and low-value clusters of self-harm prevalence. Bivariate choropleth maps were produced to visualise area-level (SA2) relationships with key Census and YMM variables.

The Hot Spot Analysis (Getis-Ord Gi*) ArcGIS tool was used to pinpoint locations of statistically significant high-value and low-value clusters of the primary outcome of interest (synthetic self-harm prevalence, irrespective of intent); to identify clusters of SA1s that represent regions of high prevalence of self-harm (in the ≥90th percentile). This Hot Spot Analysis uses the Getis-Ord Gi* statistic (*a z*-score), which indicates whether features (e.g., SA1 regions) with high values or low values (of self-harm prevalence) cluster in a specific area, by looking at each feature within the context of neighbouring features, and against all features in the dataset (i.e., the global mean) (Torok *et al.*, 2017). If a feature’s value is high, and the values for all its neighbouring features are also high (relative to the expected local sum), and that difference is too large to be the result of random chance, it is a statistically significant ‘hot spot’ (*p* < .05). The Hot Spot Analysis assigns a Gi* statistic. Clusters with a Gi_Bin of ±3 (Gi* *z*-score: <−2.58 or >+2.58) reflect statistical significance with a 99% confidence level; areas with Gi_Bin of ±2 (Gi* *z*-score: <−1.96 or >+1.96) reflect a 95% confidence level; while areas with Gi_Bin of ±1 (Gi* *z*-score: <−1.65 or >+1.65) reflect a 90% confidence level. For the current hot spot analysis, unit of analysis was SA1, outcome of interest was synthetic youth self-harm prevalence estimates (irrespective of intent) and national as opposed to state/territory cluster detection was employed; the former has been found to be more conservative with detection of suicide-related clusters (Cheung *et al.*, 2013). Spatial relationships were conceptualised as ‘contiguity edges only’, and the false discovery correction option was applied to adjust for multiple comparisons and any spatial dependencies.

## Results

### Self-harm prevalence estimates

Direct estimates of crude prevalence of self-harm by stratum, state, and sex were estimated (see Table S1 in Appendix 3). Overall estimated crude prevalence of self-harm (irrespective of intent) in preceding 12 months among Australians aged 12–17-year-olds was 8.7% (95% CI: 7.6–9.9%); non-suicidal self-harm was 8.0%, and suicide attempts was 2.4%. For all outcomes, prevalence was highest in females aged 16–17 years. Northern Territory (24.0%), Western Australia (10.0%), and South Australia (9.8%) had the highest estimated state prevalence for youth self-harm. See Zubrick *et al.* (2016) for a detailed presentation of YMM nationwide self-harm.

### Modelling

Table S2 shows results of individual- (or person-level) logistic regression modelling (see Table 1 for a description of candidate predictors). Psychological distress (based on Kessler-10 [K10] scores ≥30; odds ratio [OR] = 9.3, 95% CI = 4.8–18.1) and youth-reported major depression (Diagnostic Interview Schedule for Children [DISC-IV], see Table 1 for DISC-IV major depression description; OR = 3.9, 95% CI = 2.5–6.0) were strongly associated with self-harm, with large effect sizes (OR ≥ 4.0). Family connectedness (self-reported) was also notable, with low connectedness associated with three-fold increased odds. Other variables, including parent-reported depression, illicit substance use, alcohol use, and being bullied/cyberbullied were also associated with self-harm, but reported ORs were less than 2.5 (OR range: 1.5–2.4). The multivariable model (Table S2) had excellent discriminatory ability (area under the curve [AUC] = 0.90) and good agreement between observed and predicted numbers of self-harm cases across probability deciles; a simpler model with only three predictors (youth and parent-reported major depression combined with distress) produced an AUC of 0.88. Although there was some evidence for a univariable association between Aboriginal and/or Torres Strait Islander Status and self-harm (OR = 1.7; 95% CI: 1.0–2.9), this was attenuated by other variables and did not remain in the multivariable model.

In terms of auxiliary Census variables and individual-level self-harm (Table S3), there was evidence for associations between self-harm and area-level synthetic internalising disorders (i.e., anxiety/depression; see Appendix 4 for detailed ‘internalising disorder’ description) (highest quintile; OR = 2.2, 95% CI = 1.4–3.5); SA1-level proportion of Aboriginal and/or Torres Strait Islander youth (12–17) (OR = 1.8, 95% CI = 1.2–2.8); and a higher SA1-level proportion (≥20%) of unemployed single parent families (OR = 1.8, 95% CI = 1.2–2.8). A higher SA1-level proportion of overseas-born parents (≥30%) was associated with reduced risk of self-harm (OR = 0.7, 95% CI = 0.50–0.99), and there were also negative associations with area-level socio-economic advantage indicators (including the Index of Economic Resources [IER] and Index of Education and Occupation [IEO]; see Table S3). Similar results were found for associations with area-level self-harm. Cross-tabulations (*not shown)* for area-level (SA1-level) relationships found the following auxiliary Census variables were associated with area-level self-harm: percentage identifying as Aboriginal and/or Torres Strait Islander, low socio-economic advantage indicators (IER, low-income households), and percentage of parents born overseas. The above variables were candidates for multi-level predictive modelling for deriving synthetic self-harm estimates.

### SAE: Deriving synthetic self-harm prevalence estimates

See Table 2 for the final mixed-effects logistic regression models (with SA1-level fixed effects, and PHN-level random effects) used to derive synthetic self-harm prevalence estimates. Table 2 reports on the final multivariable models (where modelling was performed separately for each outcome and each of the candidate variables was first tested in univariable models). Table 2 only includes variables that significantly related to each outcome, and therefore, the final variable list depends on outcome. In the final models, the strongest predictor of area-level youth self-harm (irrespective of intent) was a high percentage of single parents not in the labour force (OR = 2.5; 95% CI = 2.2–2.9). Strong effects for this variable were also observed for secondary outcomes; both non-suicidal self-harm and suicide attempts, with a more modest association with suicidality (inclusive of ideation, plans or attempts). Being in an area where ≥30% parents were born overseas was associated with reduced odds of self-harm (OR = 0.7; 95% CI = 0.6–0.8), with reduced odds for both non-suicidal self-harm and suicide attempts. A higher proportion of females in an area was associated with increased odds of non-suicidal self-harm and suicidal ideation/planning, but not attempts. Compared to areas with <1% of 12–17-year-olds identifying as Aboriginal or Torres Strait Islander, increased odds of non-suicidal self-harm and suicide attempts were observed for areas with 1–10% identifying as Aboriginal or Torres Strait Islander (and decreased odds for areas with ≥10% of youth identifying as Indigenous).

**Table 2.** Effect estimates for variables used to determine model-based SA1-level predicted self-harm prevalence

Outcome & population	SA1-level variable category	OR (95% CI)	Adjusted (95% CI)*
**Self-harm aggregate (non-suicidal self-harm or suicide attempts; primary outcome)**^a^			
Percentage of 12–17-year olds	≥57% female	1.30 (1.15–1.46)	(0.9–1.8)
Percentage of 12–17-year olds	≥30% in two-parent families with both parents born overseas or in single parent household with parent born overseas	0.69 (0.61–0.77)	(0.5–1.0)
Percentage of 12–17-year olds	<1% Aboriginal and/or Torres Strait Islander Status	Reference	
	1–<10% Aboriginal and/or Torres Strait Islander Status	1.20 (1.07–1.34)	(0.9–1.7)
	≥10% Aboriginal and/or Torres Strait Islander Status	0.75 (0.64–0.89)	(0.5–1.2)
Percentage of 12–17-year-olds	≥20% in single-parent household where the parent is not in labour force	2.50 (2.17–2.88)	(1.6–3.8)
Of 12–17-year-olds	Any living in state housing	1.10 (0.98–1.23)	(0.8–1.5)
IRSAD	Highest quintile	1.42 (1.27–1.60)	(1.0–2.0)
**ICC (95% CI)**		**Est (95% CI)**	
**0.11 (0.06–0.18)**	**Level 2 variance (PHN)**	**0.39 (0.21–0.72)**	
**Non-suicidal self-harm**			
Percentage of 12–17-year olds	≥57% female	1.38 (1.22–1.56)	(1.0–2.0)
Percentage of 12–17-year olds	≥30% in two-parent families with both parents born overseas or in single parent household with parent born overseas	0.77 (0.68–0.87)	(0.5–1.1)
Percentage of 12–17-year olds	<1% Aboriginal and/or Torres Strait Islander Status	Reference	
	1–<10% Aboriginal and/or Torres Strait Islander Status	1.23 (1.10–1.39)	(0.9–1.8)
	≥10% Aboriginal and/or Torres Strait Islander Status	0.66 (0.55–0.79)	(0.4–1.1)
Percentage of 12–17-year olds	≥20% in single-parent household where the parent is not in labour force	2.27 (1.92–2.68)	(1.4–3.7)
Percentage of 12–17-year olds	≥10% in low-income households (<$52k)	0.74 (0.66–0.83)	(0.5–1.1)
Percentage of 12–17-year olds	≥70% where parent’s highest educational attainment is Year 10 or diploma/certificate	1.39 (1.21–1.60)	(0.9–2.1)
ARIA remoteness category	Average ARIA < 0.2 (major city)	Reference	
	Average ARIA 0.2–<2.4 (inner regional)	0.73 (0.61–0.88)	(0.4–1.1)
	Average ARIA ≥ 2.4 (outer regional/remote)	0.42 (0.31–0.58)	(0.2–1.1)
IEO category	Lowest quintile	Reference	
	Middle three quintiles	0.76 (0.67–0.88)	(0.5–1.2)
	Highest quintile	1.00 (0.82–1.21)	(0.6–1.8)
Proportion internalising disorder^d^	Lowest quintile	Reference	
	Middle three quintiles	0.76 (0.66–0.88)	(0.5–1.2)
	Highest quintile	0.92 (0.75–1.12)	(0.5–1.0)
**ICC (95% CI)**		**Est (95% CI)**	
**0.14 (0.08−0.24)**	**Level 2 variance (PHN)**	**0.56 (0.30–1.04)**	
**Suicide attempts**			
Percentage of 12–17-year olds	≥30% in two-parent families with both parents born overseas or in single parent household with parent born overseas	0.56 (0.45–0.69)	(0.3–1.1)
Percentage of 12–17-year olds	<1% Aboriginal and/or Torres Strait Islander Status	Reference	
	1–<10% Aboriginal and/or Torres Strait Islander Status	1.69 (1.39–2.06)	(0.9–3.1)
	≥10% Aboriginal and/or Torres Strait Islander Status	0.75 (0.56–0.99)	(0.3–1.8)
Percentage of 12–17-year-olds	≥20% in single-parent household where the parent is not in labour force	3.19 (2.53–4.03)	(1.6–6.4)
Of 12–17-year-olds	Any living in state housing	1.27 (1.03–1.55)	(0.7–2.3)
IEO category	Lowest quintile	Reference	
	Middle three quintiles	0.59 (0.47–0.75)	(0.3–1.2)
	Highest quintile	0.73 (0.50–1.06)	(0.2–2.2)
**ICC (95% CI)**		**Est (95% CI)**	
**0.24 (0.13–0.40)**	**Level 2 variance (PHN)**	**1.01 (0.48–2.15)**	
**Suicidal ideation/plans^b^**			
Percentage of 12–17-year olds	≥57% female	1.36 (1.18–1.58)	(0.9–2.1)
Percentage of 12–17-year olds	<1% Aboriginal and/or Torres Strait Islander Status	Reference	
	1–<10% Aboriginal and/or Torres Strait Islander Status	0.70 (0.60–0.81)	(0.4–1.1)
	≥10% Aboriginal and/or Torres Strait Islander Status	1.05 (0.85–1.29)	(0.6–2.0)
Percentage of 12–17-year olds	≥70% where parent’s highest educational attainment is Year 10 or diploma/certificate	1.57 (1.33–1.85)	(1.0–2.6)
Of 12–17-year-olds	Any living in state housing	1.40 (1.22–1.61)	(0.9–2.1)
IER category	Lowest quintile	Reference	
	Middle three quintiles	0.93 (0.75–1.14)	(0.5–1.7)
	Highest quintile	1.68 (1.32–2.14)	(0.8–3.5)
Proportion internalising disorder^d^	Lowest quintile	Reference	
	Middle three quintiles	1.58 (1.29–1.93)	(0.9–2.9)
	Highest quintile	1.85 (1.44–2.37)	(0.9–3.9)
**ICC (95% CI)**		**Est (95% CI)**	
**0.24 (0.14–0.38)**	**Level 2 variance (PHN)**	**1.06 (0.54–2.06)**	
**Suicidality^c^ (ideation, plans or attempts)**			
Percentage of 12–17-year olds	≥57% female	1.22 (1.08–1.38)	(0.8–1.8)
Percentage of 12–17-year olds	≥20% in single-parent household where the parent is not in labour force	1.32 (1.11–1.57)	(0.8–2.2)
Percentage of 12–17-year olds	≥10% in low-income households (<$52k)	0.67 (0.59–0.76)	(0.5–1.0)
Percentage of 12–17-year olds	≥70% where parent’s highest educational attainment is Year 10 or diploma/certificate	1.24 (1.08–1.43)	(0.8–1.9)
Of 12–17-year-olds	Any living in state housing	1.35 (1.19–1.52)	(0.9–1.9)
IEO category	Lowest quintile	Reference	
	Middle three quintiles	0.81 (0.70–0.93)	(0.5–1.2)
	Highest quintile	0.92 (0.75–1.13)	(0.5–1.7)
Proportion internalising disorder^d^	Lowest quintile	Reference	
	Middle three quintiles	1.38 (1.16–1.64)	(0.8–2.3)
	Highest quintile	1.85 (1.50–2.28)	(1.0–3.5)
**ICC (95% CI)**		**Est (95% CI)**	
**0.16 (0.09−0.26)**	**Level 2 variance (PHN)**	**0.61 (0.32–1.18)**	

*Notes*. This table only reports on the final models; therefore, the variables listed were significantly related to each outcome in univariable modelling, and as such, the final variable list in this table depends on outcome. IRSAD = Index of Relative Socio-economic Advantage and Disadvantage; IER = Index of Economic Resources; IEO = Index of Education and Occupation; SAE = small area estimation; YMM = Young Minds Matter survey; OR = odds ratio; 95% CI = 95% confidence interval; SA1 = Statistical Area Level 1; ICC = intraclass correlation coefficient; Est = estimate; PHN = Primary Health Network; ARIA = Accessibility and Remoteness Index of Australia (major cities [ARIA Score 0–≤0.20], inner regional [0.20–≤2.40], outer regional [2.40–≤5.92], remote [5.92–≤10.53], very remote [10.53–≤15.0]). For all SEIFA indices (i.e., IRSD, IRSAD, IER, IEO), high scores indicate relatively low financial disadvantage. This includes IRSD where a high score indicates a relative lack of disadvantage and IRSAD where a high score indicates a relative lack of disadvantage and greater advantage overall.

a Self-harm with or without suicidal intent (non-suicidal self-harm or attempts) in the past 12 months (primary outcome).

b Ideation/plans: suicidal ideation or planning only in the past 12 months (suicide attempts excluded from variable).

c Suicidality: suicidal ideation, plans or attempts in the past 12 months.

d Synthetic small area estimation (SAE) of internalising disorder prevalence (or depression / anxiety disorder prevalence), as based on a previous Young Minds Matter analysis (see Appendix 4).

* Adjusted 95% CIs were derived by inflating the standard errors by a factor of 3.

In terms of socio-economic and housing variables, areas with any state housing (i.e., low-cost rental housing provided by state governments) had increased odds of suicidal ideation/plans and attempts (OR range = 1.3–1.4). Being in the highest quintile of economic advantage (based on the Index of Relative Socio-economic Advantage and Disadvantage [IRSAD]) was associated with increased odds of self-harm (irrespective of intent) (OR = 1.4; 95% CI = 1.3–1.6), while being in the highest quintile of the IER was associated with increased odds of ideation/plans (OR = 1.7; 95% CI = 1.3–2.1), relative to the lowest quintile (Table 2). Compared to the lowest quintile of opportunity (IEO index), areas classified in the middle three quintiles had reduced odds of non-suicidal self-harm and suicide attempts (OR range: 0.6–0.8). In terms of internalising disorders (anxiety, depression), odds of suicidal ideation/plans increased with increasing synthetic internalising disorder prevalence. Intraclass correlation values (see Table 2) indicated clustering of SA1-level prevalence estimates within PHNs, with point estimates ranging from 0.11 (self-harm irrespective of intent) to 0.24 (suicidal ideation/plans and attempts). Clustering was further explored using ArcGIS mapping tools (see below).

### Mapping and clustering analyses

**Figure 1.**
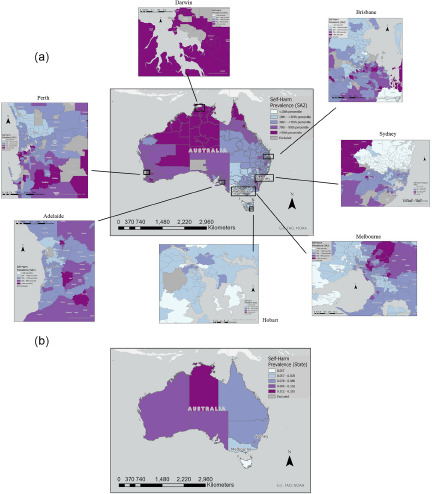
(a) Distribution of Statistical Area Level 2 (SA2) synthetic, 12-month self-harm prevalence estimates (2019), and (b) State-level synthetic, 12-month self-harm prevalence estimates (2019), Australian-wide. The primary outcome is shown in these maps, i.e., self-harm irrespective of intent. Capital cities (zoomed in images) are presented in Figure 1a.

Figure 1(a–b) shows Australian-wide maps for synthetic SAEs of youth self-harm prevalence estimates in the past 12 months (for 2019). Overall, there was large variability in prevalence estimates across Australia, for non-suicidal and suicidal self-harm. High density areas (i.e., areas with high density of self-harm cases per square km; not shown in Figure 1) were located in the capital cities and some larger regional towns in each state and territory.

**Interpretation note.** The choropleth maps show the distribution of youth self-harm prevalence in each SA2 (i.e., size of suburbs within cities) and state, where ‘Dark Purple’ indicates higher prevalence (i.e., above the 90th percentile), and ‘Light Blue’ indicates lower prevalence of self-harm (i.e., below the 10th percentile). Excluded map regions (in grey) indicate missing data or regions with low quality data (5% of total SA1s across Australia).

**Figure 2.**
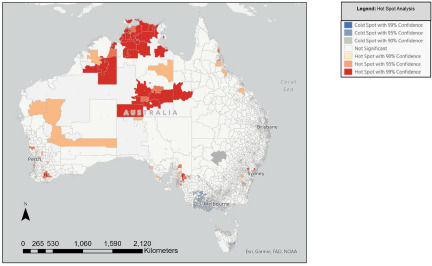
ArcGIS Hot Spot Analysis (Getis-Ord Gi*) of statistically significant clusters of youth self-harm prevalence estimates (2019), SA1.

Nationwide clustering analyses (Fig. 2) showed statistically significant clustering of regions (i.e., ‘hot spots’) with high synthetic prevalence of youth self-harm (with 90–99% confidence) across metropolitan, inner/outer regional, and remote parts of Western Australia; inner/outer regional and remote parts of Northern Territory; inner/outer regional and metropolitan parts of North and Central Queensland; metropolitan and inner/outer regional areas of New South Wales (particularly outer Western Sydney); Eastern metropolitan areas of Melbourne; and outer South-Eastern regions of Adelaide. ‘Cold spots’ (i.e., areas of statistically significant clustering of low self-harm prevalence) were found in outer Western Melbourne regions, northern Sydney, southern midland Tasmania, and the Gold Coast. There was minimal clustering detected in other parts of Australia. Supplementary analyses with secondary outcomes found non-suicidal self-harm clustering throughout Northern Territory, Western Australia, North Queensland, outer Western Sydney, Eastern Melbourne and outer Adelaide, whereas suicide attempt clustering was predominantly in East Coast regions. Supplementary analyses should be interpreted with caution due to sparse suicide attempt numbers in the YMM survey for Northern Territory, metropolitan Tasmania, and rural Western Australia.

**Interpretation note.** The ArcGIS Hot Spot Analysis map shows statistically significant high-value and low-value SA1-level clusters of the outcome of interest (self-harm prevalence) with 90–99% confidence. The ‘Dark Red’ regions indicate ‘hot spots’ of statistically significant clustering of high self-harm prevalence (with 99% confidence), the ‘Dark Blue’ regions indicate ‘cold spots’ of statistically significant clustering of low self-harm prevalence (with 99% confidence), whereas ‘White’ regions are areas with no evidence of self-harm clustering.

### Bivariate maps (self-harm and risk/protective factors)

**Figure 3.**
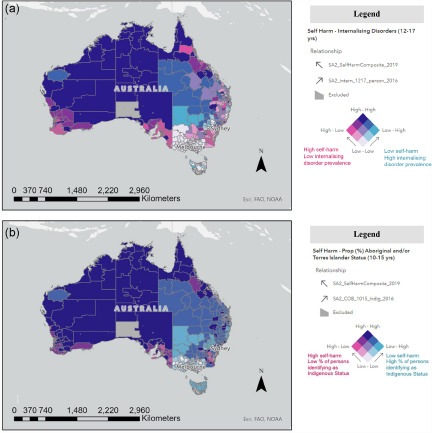
(a) Bivariate choropleth map of Statistical Area Level 2 (SA2-level) association between SAE self-harm prevalence (2019) and prevalence of internalising disorders, Australian-wide. (b) Bivariate choropleth map of Statistical Area Level 2 (SA2-level) association between SAE self-harm prevalence (2019) and proportion of Aboriginal and/or Torres Strait Islander people (Census 2016), Australian-wide.

**Figure 4.**
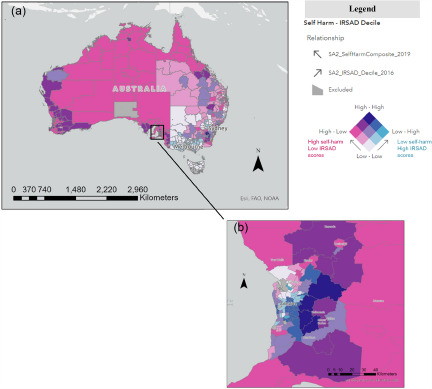
(a) Bivariate choropleth map of Statistical Area Level 2 (SA2-level) association between SAE self-harm prevalence estimates (2019) and IRSAD decile (Census 2016), Australian-wide, and (b) wider Adelaide region, South Australia (Zoomed in map - area indicated by black box in Figure 4a). The primary outcome is shown in these maps, i.e., self-harm irrespective of intent.

Relationships with key factors from Census and YMM data were further explored using bivariate mapping. For all factors, area-level associations with synthetic youth self-harm differed geographically across the nation (in size and direction), including social determinants and psychological variables. Associations between area-level internalising disorder prevalence (Fig. 3a) and area-level proportion of Aboriginal and/or Torres Strait Islander people (Fig. 3b) with self-harm were generally positive. These positive associations were strongest in self-harm cluster regions in Northern Territory, Western Australia, and North Queensland (see ‘Dark Blue’ regions, i.e., strong positive correlations in Fig. 3a–b). There were sections, however, along central inland New South Wales which demonstrated high SA2 internalising disorder prevalence but low self-harm prevalence (‘Light Blue’ regions, i.e., strong negative correlations in Fig. 3a) and vice versa of low internalising disorders but high self-harm prevalence in pockets of most capital cities and some regional cities (e.g., Mackay, Townsville, Newcastle) (‘Dark Pink’ regions in Fig. 3a). This highlights the complexity and variability of the relationship between self-harm and mental disorders. Associations between self-harm and proportion of parents born overseas and the IRSAD decile were generally negative (i.e., low socio-economic advantage, high self-harm; Fig. 4), where associations were strongest in self-harm cluster regions in Northern Territory and Western Australia. For some metro areas, however, these relationships were reversed, where high IRSAD deciles (or high socio-economic advantage) was associated with high self-harm prevalence (‘Dark Blue’ regions) in most capital cities (Fig. 4). On a general note, there was more variability in the size and direction of associations between self-harm and variables of interest in metro versus regional Australia.

**Indicator definition:** Synthetic Young Minds Matter (YMM) estimate of SA2-level prevalence of internalising disorders (e.g., major depression, anxiety disorders) among young people aged 12–17 years (see Appendix 4).**Indicator definition:** SA2-level proportion of people identifying as Aboriginal and/or Torres Strait Islander (aged 10–15 years), derived from 2016 Census data. **Interpretation note:** On these maps, dark blue colouring represents areas where both self-harm and the factor of interest have higher prevalence (i.e. a strong positive association). Associations between area-level internalising disorder prevalence (Fig. 3a) and area level proportion of Aboriginal and/or Torres Strait Islander people (Fig. 3b) with self-harm were generally positive. Please note, just as there are differences between areas, there are variations, and sometimes substantial variations, within an area. This means that the same outcome does not apply to everyone living in the named areas. Also, identifying communities whose residents are not faring as well as others, may be seen as stigmatising. However, the purpose is to highlight the extent of disadvantage and relationships with self-harm, to provide evidence upon which community members and decision-makers can rely, and which can underpin advocacy for change.

**Indicator definition:** SA2-level Index of Relative Socio-economic Advantage and Disadvantage (IRSAD), derived from 2016 Census data. High IRSAD scores indicate relatively low financial disadvantage. **Interpretation note:** Socio-economic status and self-harm largely showed strong negative relationships (i.e., low socio-economic advantage and high self-harm prevalence) across the nation (‘Dark Pink’ areas in map). However, most capital cities showed reversed pockets, i.e., areas of high socio-economic advantage and high self-harm prevalence.

## Discussion

This was the first Australian-wide study to examine regional variability of youth self-harm and related risk and protective factors. Overall, there was large geographic variation in synthetic youth self-harm prevalence across the nation, supporting Hypothesis 1. This was the case for primary and secondary outcomes (non-suicidal and suicidal self-harm). Northern Territory Western Australia, and South Australia had the highest estimated state prevalence, somewhat consistent with previous suicide incidence studies (Qi *et al.*, 2012; Robinson *et al.*, 2016b).

Significant clustering of youth self-harm prevalence (with 99% confidence) was detected across metropolitan, regional and remote parts of Western Australia, Northern Territory, Queensland, Victoria and New South Wales, supporting Hypothesis 2. This is consistent with international studies which show self-harm clustering spanning the urban–rural continuum (Congdon, 2012; Lin *et al.*, 2019; Rosychuk *et al.*, 2016), but at odds with studies reporting more prominent self-harm clustering in urban areas (Corcoran *et al.*, 2007; Hempstead, 2006; Torok *et al.*, 2017); the latter discrepancy is likely explained by the use of self-report community-based data (in the current study) versus hospital record data in the cited studies. When comparing clustering patterns for non-suicidal and suicidal self-harm, the current study found suicide attempt clustering was more predominant in East Coast regions, whereas non-suicidal self-harm clustering was relatively spread out across the country. These supplementary analyses, however, should be interpreted with caution due to overall sparse suicide attempt (YMM) numbers in West Coast states.

In individual-level modelling, poor mental health (high psychological distress and major depression) was the strongest predictor of youth self-harm. This is indicative that self-harm is largely explained by the mental health of young people (albeit not in a deterministic way), consistent with the wider self-harm and suicide literature (Carter *et al.*, 2003; Inder *et al.*, 2014; Soole *et al.*, 2015). In the final area-level model, the strongest predictor of youth self-harm was area-level percentage of single unemployed parents, whereas areas with high percentages of families with overseas-born parents was associated with reduced odds. Previous Australian suicide studies (Law *et al.*, 2014) have shown similar results in second-generation migrant young adults (i.e., born in Australia but with at least one parent from overseas). It is postulated that this lowered suicide risk could be related to the healthy migrant effect (Kennedy *et al.*, 2015), where persons who meet Australia’s immigration rules, and who choose to migrate, are on average healthier and better educated than native-born. Growing up in bi- and multicultural neighbourhoods may also be protective against self-harm and suicide, providing youth with a bigger repertoire of skills and attitudes (Law *et al.*, 2014; Neeleman *et al.*, 2001). Others caution against such interpretations, as there may be self-harm underreporting in these communities related to stigma and shame (Pham *et al.*, 2021).

Small area relationships were further explored using bivariate mapping. All associations with self-harm differed geographically across the nation, emphasising the complexity of these relationships. This included social determinants (socio-economic indices) as well as psychological variables (internalising disorders), providing no support for Hypothesis 3. For example, for socio-economic indices, there was an overall negative association with self-harm. Within most capital cities, however, relationships were reversed. This contrasts with the wider literature showing negative, linear self-harm-socio-economic associations (Fontanella *et al.*, 2015). Some international small-area studies show non-linear relationships with socio-economic status (Congdon, 2012; Hsu *et al.*, 2015) and a recent Australian study showed the greatest growth in youth self-harm hospital presentations was in socio-economically advantaged areas (Sara *et al.*, 2022). The study authors (Sara *et al.*, 2022) postulated that this could be related to the ever-growing pressures for educational attainment among families with higher incomes. Non-linear relationships were also demonstrated in the modelling for proportion of the population identifying as Aboriginal and/or Torres Strait Islander youth, where increased odds of non-suicidal self-harm and suicide attempts were observed for areas with 1–10% identifying as Indigenous youth (and decreased odds for areas with more than 10%). Spatial patterns in the bivariate maps (see Fig. 3b) further illustrated that these non-linear relationships were particularly prominent in surrounding urbanised areas of Sydney, Melbourne and Brisbane. Outside of these areas, there was largely a strong positive relationship between self-harm prevalence and proportion identifying as Indigenous Status (see Fig. 3b). These bivariate spatial patterns are largely consistent with previous Australian adult studies (Qi and Hu, 2009; Cheung *et al.*, 2014; Qi *et al.*, 2014) where proportion of Aboriginal and Torres Strait Islander peoples was shown to be an important factor, associated with spatial patterns for male suicides.

### Strengths and limitations

The current study is significant in addressing a public health gap, generating youth self-harm prevalence estimates at geographic levels not typically available. Most previous spatial studies report on suicide deaths or self-harm hospitalisation data, and predominantly in adult samples. Most have also conducted analyses at broader spatial aggregations (i.e., local government areas), which may have limited translation utility for local-level stakeholders. All modelling incorporated high-quality survey and Census data, and our synthetic self-harm estimates were validated using direct estimates of suicides from the National Mortality Database.

Synthetic estimates are the best available small area data that can be used for service planning. However, a key limitation is that firstly, these are not direct estimates, but rather are based on a model with a set of assumptions (see Appendix 1). Where these assumptions are violated, model predictions may not be accurate. It is not possible to test these assumptions without detailed small-area data on actual numbers of self-harm cases. Secondly, estimates were prepared using 2013–14 YMM (nationally representative) survey data, and updated to 2019 using population data. The accuracy of synthetic estimates could be impacted by this, as we could not incorporate recent trends of increasing self-harm hospitalisations (Bastiampillai *et al.*, 2021; Sara *et al.*, 2022) and other recent population changes (e.g., impacts of natural disasters, COVID-19 pandemic). Thirdly, YMM survey coverage was low in certain states/territories (Northern Territory, Tasmania, Western Australia) which may have impacted the accuracy of suicide attempt estimates in regional and remote areas. Finally, whilst the variables included in the current modelling were comprehensive, it was not an exhaustive list where some other important variables related to youth-self-harm could not be included, such as externalising/risky behaviours (apart from alcohol/substance use).

### Future research and implications

Nationwide spatial analyses provide a clear rationale for where future youth self-harm and suicide prevention efforts should be prioritised across Australia. This included youth self-harm clustering in metropolitan and regional areas across Western Australia, Northern Territory, Queensland, Victoria, and New South Wales. Spatial analyses can only provide an indication of what *may* be happening locally. This data gap should drive efforts towards establishing local partnerships with relevant data custodians (Torok *et al.*, 2017), making real-time data more accessible in these regions. This study also identified key variables that should inform targets for future prevention initiatives, including targets for further research inquiries. This includes continued concerted efforts for: improving young Australians’ mental health particularly in rural and under-served areas; policy measures to support the employment outcomes of single parents; as well as further investigations into the potential protective elements of living in a migrant-rich community. In terms of findings related to young people identifying as Aboriginal and/or Torres Strait Islander, further research is required to better understand the current non-linear findings and broader contextual effects, including a follow-up study of adverse and protective experiences of Indigenous youth is warranted, one which is led by Indigenous researchers and community leaders.

## Conclusions

There was overall large variability in youth self-harm prevalence estimates (non-suicidal and suicidal) across Australia. Nationwide analyses identified local areas in each state and territory where future youth self-harm and suicide prevention efforts should be prioritised. Mental ill health, parent unemployment, and being born to Australian-born parents were key risk factors of youth self-harm in multi-level modelling. Bivariate mapping showed all associations with self-harm differed geographically across Australia, emphasising the complexity of self-harm relationships. Current findings should assist Australian policy makers, service planners, and commissioners with planning and implementing regionally appropriate youth self-harm and suicide prevention initiatives and interventions, and in turn, ultimately help target resources where they are likely to have the greatest impact on reducing youth self-harm and suicide.

## Supporting information

Hielscher et al. supplementary material Hielscher et al. supplementary material

## Data Availability

Data from the Australian Youth Self-Harm Atlas are available via the National Suicide and Self-Harm Monitoring System Website (https://www.aihw.gov.au/suicide-self-harm-monitoring/data/geography/youth-self-harm-atlas), hosted by the Australian Institute for Health and Welfare (AIHW). This online data dashboard includes (1) primary outcome (self-harm) prevalence choropleth maps; (2) secondary outcome (non-suicidal self-harm, suicide attempts, ideation/plans) prevalence choropleth maps and (3) bivariate choropleth maps visualising the association between self-harm and key risk/protective factors. Maps are presented at various spatial units, allowing for flexibility in the presentation of the data. Young Minds Matter data are also available to interested readers through the Australian Data Archive: https://dataverse.ada.edu.au/dataset.xhtml?persistentId=doi:10.4225/87/LCVEU3.

## Supplementary material

The supplementary material for this article can be found at https://doi.org/10.1017/S2045796024000301.
