# Supplementary material for: Australian Youth Self-Harm Atlas: spatial modelling and mapping of self-harm prevalence and related risk and protective factors to inform youth suicide prevention strategies
Source: Epidemiol Psychiatr Sci. 2024 Sep 9;33:e34. doi: 10.1017/S2045796024000301 (PMC11450422; doi:10.1017/S2045796024000301)
Supplement: Hielscher et al. supplementary material [file S2045796024000301sup001.docx]

# SUPPLEMENTARY MATERIALS

Low self-harm

High IRSAD scores

Appendix 1. Additional Information on Modelling, Assumptions, and Limitations

Appendix 2. External Validation of Synthetic Self-Harm Prevalence Estimates

## Appendix 3. Additional Results Tables and Figures

Appendix 4. Synthetic Internalising Disorder Estimates

Please note that Appendix 1 (and all other appendices) refer to both 2011 and 2019 datasets. 2011 Census data was used in modelling (baseline population data at the time of the YMM survey). Equivalent 2016 Census data with 2019 estimated resident population (ERP) count data were used to derive synthetic youth self-harm prevalence estimates for 2019.

**Appendix 1. Additional Information on Modelling, Assumptions, and Limitations**

**Modelling Steps**

The Young Minds Matter (YMM) survey was conducted between May 2013 and April 2014; data were available for 2,578 youth from 540 SA1s. We assume the direct self-harm prevalence estimates are accurate for large geographical areas (e.g., PHN) and stable over time. The sample was insufficient to provide reliable direct small area prevalence estimates. The goal of modelling was to produce SA1-level prevalence estimates suitable for aggregating up to higher geographical areas and exploratory spatial analyses rather than reporting SA1-level prevalence estimates with measures of precision.

Outcomes of interest were modelled using generalised linear modelling, specified using the binomial family and the logit link (using the following specifications/equations below):

$$Y_{i}\sim binomial, \left( {n_{i}, \pi}_{i} \right)$$

$$\mathrm{logit} \left( \pi_{i} \right)=\frac{\ln(\pi_{i})}{1-ln(\pi_{i})}= \boldsymbol{\beta X}_{i}^{T}$$

Where $Y_{i}$ is the observed outcome for unit *i*, assumed to follow a binomial distribution with probability $\pi_{i}$ and $n_{i}$ is the number of observations in unit *i.* For individual-level models $n_{i}=1$*.*

The log-odds of the expected probability of a positive outcome is assumed to be linearly related to a vector of explanatory variables $\boldsymbol{X}$ with regression coefficients $\boldsymbol{\beta}$**.**

Key modelling steps outlined below.

### Step 1: Self-harm prevalence estimates

Estimated prevalence of self-harm was derived using the “survey” suite of commands in the Stata statistical software package (version 15). Data were “survey set”, defining strata, Statistical Area Level 2 (SA2) as the primary sampling unit, and clustering of households within SA1s, and SA1s within SA2s. Individual level sampling weights were specified using the “YouthWeightC” variable in the YMM survey.

For each subpopulation of interest, prevalence estimates were obtained. Direct estimates with exact 95% confidence intervals were presented overall, by age group and sex, and by state and stratum. Crude prevalence estimates for SA1 were used in modelling to derive synthetic estimates.

### Step 2: Associations between variables of interest and self-harm

Associations with outcomes of interest were assessed using all available data. Associations were firstly assessed in person-level (or individual-level) models before building area-level models. Exposure variables of interest as predictors of self-harm were identified a priori, based on a literature review and expert knowledge. A causal diagram was constructed in which postulated inter-relationships between variables were depicted graphically.

### Step 3: Individual-level self-harm model

Eligible variables with p-values <.20 in univariable models were entered and tested in a multivariable model (where individual-level self-harm was the outcome variable). A stepwise backwards selection process was applied whereby the variable with the highest p-values was removed at each step until all remaining variables were significant at the 5% level. Excluded variables were re-entered and tested in the final model. Akaike’s Information Criteria (AIC) was used to select the best-fitting final base model. The discriminatory ability of the model was assessed using the area under the receiver operator characteristic or ROC curve (also referred to as ‘AUC’), and goodness of fit was assessed using Hosmer-Lemeshow’s goodness of fit test and inspecting the distribution of observed to predicted values by probability decile. Variables derived from auxiliary data were entered and tested in the final model, but none of the candidate variables improved model fit or discriminatory ability. Estimates for the final individual-level self-harm model were obtained after fitting the logistic regression model within Stata’s “survey” suit of commands, to ensure that standard errors were adjusted to account for the complex survey design.

### Step 4: Individual-level self-harm model using only auxiliary data

To better understand possible relationships with area-level data, logistic regression modelling was repeated using only auxiliary Census data (where individual-level self-harm was the outcome variable). Based on the individual-level model, variables of particular interest were those that would capture area-level associations (e.g., SA1-level proportion of Aboriginal and/or Torres Strait Islander peoples and area-level/SA1-level self-harm). Because individual variables measuring psychological status (distress, depression) were the strongest predictors of self-harm and there was no auxiliary data available to capture this directly, the synthetic estimate of SA1-level prevalence of internalising (or depression/anxiety) disorders (derived from previous analyses of the YMM youth data; see Appendix 4) was tested in this model. This variable was categorised into quintiles and explored further after collapsing the middle three categories.

Associations with auxiliary variables were assessed for both continuous and categorical versions of variables of interest. For most continuous variables, the assumption of linearity of the logit was violated, so categorical variables were used in final modelling.

### Step 5: Exploring variability and clustering

The data had a multilevel structure with households nested within SA1s, nested within SA2s, nested within SA3s, nested within Primary Health Networks (PHNs) within states. Clustering of the outcome measure (self-harm) was explored by comparing models fitted with/without random effects. The intraclass correlation coefficient (ICC) is a measure of clustering. This was assessed in null models (i.e., no covariates), and in the final models with covariates. Clustering was assessed in both the individual-level and area-level models.

**Synthetic Estimation Steps**

Small area estimation (SAE) techniques utilise statistical modelling to produce model-based “synthetic” estimates for both in-sample and out-of-sample areas (Rao & Molina, 2015). The number of YMM survey respondents per SA1 across Australia was small (only ~1% SA1s were sampled in the YMM survey). Hence, any “design-based” estimate of self-harm prevalence would be highly imprecise and of limited use. “Model-based” estimates borrow strength and are useful to provide prevalence estimates (either unadjusted or adjusted for covariates). SAE techniques utilise out-of-sample predictions to derive model-based “synthetic” estimates for areas with no survey respondents. They utilise measures that are common to both the measured population and the unmeasured population.

Auxiliary Census data from the ABS were available for all SA1s for 2011 and 2016. Because we lose information by aggregating data, modelling was performed using SA1-level ABS data. Hierarchical generalised linear models make efficient use of available data by borrowing strength across areas, providing more stable estimates. Area-level predicted probabilities are shrunken (smoothed) towards the overall mean. We would expect model-based fixed effect estimates to be over-dispersed, while estimates from multilevel models to be shrunken relative to the empirical distribution. Hence, smoothed prevalence estimates obtained from multilevel modelling were benchmarked using direct prevalence estimates derived from the YMM survey. We used the estimated population of 12-17 year olds based on the 2011 census with the YMM direct estimates to derive the SA1-level estimated count for each outcome in the sampled areas for use in modelling; the inflated sample size was 24,322. We acknowledge that this will result in overly precise estimates for model coefficients; post hoc adjusted 95% confidence intervals were produced by multiplying the standard errors by three (~√(24322/2578)).

Key steps for small area estimation are outlined below.

***Step 1: Small area estimation (SAE): Area-level model using only auxiliary data and derivation of synthetic self-harm estimates***

Prevalence estimates of each outcome of interest were obtained for different hierarchical levels (SA1 [Statistical Area level 1], SA2, SA3, SA4, PHN [Primary Health Network], state) from the original individual-level survey data, taking account of the complex sampling scheme. From the estimated point prevalence and number of 12-17 year olds in each SA1 (from the 2011 Census), a crude estimated count for the number of 12-17 year-olds with each outcome for each SA1 was derived for each SA1 with survey data. The effects of auxiliary variables and clustering of prevalence at different hierarchical levels was explored by fitting mixed-effects logistic regression models.

Predictor variables of interest (for SAE modelling) were identified based on literature review, expert opinion, and observed associations with individual-level self-harm in the YMM survey data (see previous modelling steps with auxiliary variables on pg. 2-4). Correlations between candidate variables were assessed and noted. Separate multivariable models were built for each outcome (primary variable: self-harm irrespective of intent; secondary variables: non-suicidal self-harm, suicide attempts, suicidality, ideation/plans), using one candidate variable from each domain. Candidate variables were screened in univariable logistic regression models. For each domain showing an association with the outcome, one candidate variable was selected (based on quality of the variable and strength of association) and entered into a multivariable model. Variables were dropped sequentially based on the highest p-value, before being re-entered and tested. Consistency of associations were checked in models with different random-effect level specification (none, SA3, SA4, PHN). After considering the distribution of observed data (number of observations per cluster), clustering across different hierarchical levels (ICC), and agreement between aggregated model-based predictions and survey-based estimates. Final models were selected to include predictors with consistent effects across model specification and using model fit criteria (deviance). Two-level grouped logistic regression models were specified using SA1 as the unit of analysis and a random intercept for PHN. The final model specifications and equations are outlined below:

**Final model specifications and equations**

The unit of analysis for the final model was SA1. The expected count of the number of cases within each SA1(*i*) was derived from the direct estimates of the proportion from the YMM survey and the denominator ($n_{i}$) was the total number of 12-17 year olds in the SA-1 based on the 2011 census.

Single level models were fitted for each outcome as described above. These were expanded to incorporate random intercepts for each level-2 unit, $\upsilon_{j}$.

$\mathrm{logit} \left( \pi_{ij} \right)= \boldsymbol{\beta X}_{ij}^{T}$ +$\upsilon_{j}$

The PHN was chosen for the level-2 unit because there were an adequate number of SA1s within most PHNs and this choice provided geographical coverage across the majority of the country. We are assuming that SA1s nested within each PHN are more similar to each other than to those in different PHNs.

The log-odds of the expected probability of a positive outcome is assumed to be linearly related to a fixed effects (i.e. a vector of explanatory variables $\boldsymbol{X}$ with regression coefficients $\boldsymbol{\beta}$) plus the level-2 random effects ($\upsilon_{j}$)

The model-based predicted probability for each outcome was derived as:

$$\pi_{ij}=\frac{\exp(\boldsymbol{\beta X}_{ij}^{T} +\upsilon_{j})}{1+\exp(\boldsymbol{\beta X}_{ij}^{T} +\upsilon_{j})}$$

For each PHN (*j*), assuming there were *M* SA1-level units (*i*), the initial model-based estimate was derived by summing the predicted counts (numerators) and census counts (denominators):

$$\pi_{j}=\frac{\sum_{i=1}^{m} {n_{i}\pi}_{i}}{\sum_{i=1}^{m} n_{i}}$$

Model-based predicted counts and prevalences for each SA1 were derived for 2011 incorporating both fixed and random effects (where this mixed effect modelling produced smoothed or shrunken estimates). In addition, model coefficients and estimated random effects were used with the SA1-level auxiliary variables derived from the 2016 Census to derive predicted prevalence estimates for each outcome for each SA1 in 2019, incorporating the estimated resident population (ERP) for 2019. The predicted counts (number of events) and Census totals (number of 12-17 year olds) in 2011 were aggregated within PHNs to derive the model-based predicted PHN-prevalence for each outcome. These were compared to the YMM prevalence estimates to derive benchmark factors (YMM prevalence/PHN estimated prevalence). Benchmarked prevalences were derived for each SA1 for each year of interest by multiplying the benchmark factor by the model-based predicted prevalence. The benchmarked count for each SA1 for each year was derived as the benchmarked-prevalence multiplied by the ERP of 12-17 year olds for 2011 and 2019.

These benchmarked estimates were aggregated over each area unit of interest (SA2, SA3, SA4, PHN, state) to derive the total count (numerator), while the denominator was the total of the relevant ERP (for areas with non-missing predictions). The benchmarked prevalence of each outcome for each level was obtained by dividing the aggregated estimated case count by the aggregated population count. Areas without predictions did not contribute to the numerator or denominator. The distribution of benchmarked SA1-level prevalence SAEs was presented graphically using histograms with 2011 and 2019 estimates overlaid. For each outcome (primary and secondary), case density measures (i.e., cases per square km) were derived as the benchmarked count divided by SA1 area for each outcome and for each year (2011, 2019).

### Step 2: Validation using external suicide dataset (NMD)

Suicide death data from the National Mortality Database (NMD) (available for all SA2s in Australia) was incorporated to validate the synthetic self-harm estimates. Associations between SA2-level small area estimates (SAEs) of youth self-harm and crude suicide rates were assessed. Data comprised SA2-level (ASGS: 2016 version) crude annual adult suicide rates (per 100,000 population) averaged over 5-year periods from the NMD. These were averaged to derive SA2-level average annual rates for a 10-year period (2010–2019). Both mean suicide rate and SAE data were square-root transformed to achieve an approximately normal distribution suitable for use in modelling. For SA2s with sufficient data and an estimated minimum of 20 young people (12-17 years), the relationship between crude suicide rates and SA2-level SAEs of youth self-harm prevalence was assessed by inspecting scatter plots, assessing correlations, and fitting linear regression models. Separate models were fitted for 2011 and 2019. See Appendix 2 for further details about this validation step, and its results.

**Assumptions and Limitations – Small Area Estimation**

The major limitation of synthetic estimates is that they are not direct estimates from the survey for individual regions. They are based on a model with a set of assumptions. Where these assumptions are violated, the predictions of the model will not be accurate. It is not possible to test these assumptions with the data that is available. While synthetic estimates are the best available small area data that can be used for service and program planning and delivery, the actual number of cases of self-harm in any small area can vary from the model prediction if there are unique factors of that area that are not included in the model.

#### **Key modelling assumptions**

Synthetic estimates are based on the assumption that prevalence of self-harm in any small area can be determined based on knowing the socio-demographic characteristics of the area, and that the relationship between socio-demographic characteristics and prevalence of self-harm does not drastically vary between broad geographic areas. There is some basis for making this assumption as the prevalence of self-harm has remained relatively stable across time and between developed countries where measurements have been made (although there is evidence of recent rising trends of self-harm hospitalisations, among Australians aged 15-19 (Bastiampillai *et al.*, 2021; Sara *et al.*, 2022)). It is not possible to test these assumptions without the availability of detailed small area data on actual numbers of cases. For instance, if a particular community happens to have an effective self-harm or suicide prevention program that is not available elsewhere, or if there is some environment factor in the local environment that is a risk factor for self-harm/suicide, these factors would not be captured by the synthetic estimation approach.

***Other modelling considerations***

We assume that the relationships between auxiliary variables (that measure socio-demographic characteristics of the area) and outcomes are consistent across areas and over time. Model coefficients and estimated random effects were used with the SA1-level auxiliary variables derived from the 2016 Census to derive predicted prevalence estimates for each outcome for each SA1 in 2019, incorporating the estimated resident population (ERP) for 2019. Area-level predicted probabilities are shrunken (smoothed) towards the overall mean. We assume that estimates can be reliably reflated using a PHN-level benchmarking factor which will ensure that aggregated estimates will match the PHN-level direct prevalence estimates. The predicted counts (number of events) and Census totals (number of 12-17 year olds) in 2011 were aggregated within PHNs to derive the model-based predicted PHN-prevalence for each outcome. These were compared to the YMM direct prevalence estimates to derive benchmark factors (YMM prevalence/PHN estimated prevalence). Benchmarked prevalences were derived for each SA1 for each year of interest by multiplying the benchmark factor by the model-based predicted prevalence. The benchmarked count for each SA1 for each year was derived as the benchmarked-prevalence multiplied by the ERP of 12-17 year olds for 2011 and 2019. These were aggregated over each area unit of interest (SA2, SA3, SA4, PHN, state) to derive the total count (numerator), while the denominator was the total of the relevant ERP (for areas with non-missing predictions). The benchmarked prevalence of each outcome for each level was obtained by dividing the aggregated estimated case count by the aggregated population count. Areas without predictions did not contribute to the numerator or denominator.

#### **1. Region size**

Statistical Areas Level 1 (SA1s) are geographic areas built from whole Mesh Blocks and are designed to maximise the geographic detail available for Census of Population and Housing data. There are 61,845 SA1 regions covering the whole of Australia without gaps or overlaps, where SA1s generally have a population of 200 to 800 people, and an average population of about 400 people. SA2s are medium-sized general-purpose areas built up from whole SA1s. Their purpose is to represent a community that interacts together socially and economically. There are 2,473 SA2s covering the whole of Australia without gaps or overlaps, generally have a population between 3,000 and 25,000 with an average of about 10,000 people.

While SA1s and SA2s have been designed to have approximately similar population sizes, there are some SA1s/SA2s in Australia that have no population and some that have very small populations. The accuracy of synthetic estimates declines as regions become smaller, because there is more individual variation in the Census data. Where there is no population in the SA1 (or SA2) the synthetic estimates of youth self-harm prevalence must be assumed to be zero. For some SA1s/SA2s with very small populations it was not possible to calculate synthetic estimates because there were insufficient numbers to populate Census tabulations for that area (4.4% of total SA1s) or there was incomplete Census information on covariates that were to be incorporated into self-harm predictive modelling (0.65% of total SA1s).

#### **2. Cohort ageing**

These small area estimates have been prepared using data from the YMM survey, which was collected between May 2013 and April 2014, and applied to small area socio-demographic data taken from the 2016 Census and updated to June 2019 using Estimated Resident Population (ERP) data, supplied by the ABS.

Children who were aged 12-17 years in 2016 would be aged 18-23 years in 2022. Hence none of the adolescents in the target age range (of 12-17 years) in the 2016 Census would still be in the target age range now. With birth rates remaining relatively stable over this period, the numbers of 12-17 year-olds in 2016 compared with 2022 display steady population growth at broad geographic levels. However, at a small area level, children who were 12 years old in 2016 and who would be 18 years old in 2022 may be living in different geographic locations, compared with children who are 12 years of age in 2022. In addition, there is no information to say whether the 12-17 year-olds living in a small geographic area in 2016 share characteristics with the 12-17 year-olds who are living in that area in 2022. For example, contemporary factors that may influence youth mental health and self-harm could not be accounted for, including recent changes in technology use, increasing effects of climate change (and other recent disasters e.g., COVID-19), and changes to the service landscape (e.g., increased number of headspace services).These cohort effects can have a significant impact on the accuracy of small area estimates that are prepared for specific age groups.

The ABS provided two Estimated Resident Population figures as at 30 June 2019 — for 4-11 year-olds and for 12-17 year-olds. These ERP figures were used to calculate population growth for small areas between 2016 and 2019. However, no information is available as to whether there have been changes in the distribution of socio-demographic characteristics since 2016, and no information is yet available about population changes between 2016 and 2022. Such information could not be incorporated into the current modelling.

**Additional Information – Risk and Protective Factors of Interest**

Variables of interest as predictors of self-harm were identified a priori, based on a review of relevant literature (Hawton, Saunders, O’Connor 2012; Cantor & Neulinger, 2000; Hill et al., 2020; Too et al., 2017; Inder et al., 2014; Torok et al., 2017) and expert knowledge.

Variables of interest as predictors of self-harm included:

**YMM data**

Person-level variables:

- - 1. Demographics: age, sex, Aboriginal and/or Torres Strait Islander Status, country of birth
    2. Psychological assessment screens:
  - Kessler-10-item psychological distress scale (K-10)
  - Diagnostic Interview Schedule for Children (DISC-IV) major depression (youth-reported and parent-reported, past 12 months). ‘Parent reported’ is the parents’ assessment of their child’s mental health.
  - Self-esteem – Adolescent Self-Esteem Questionnaire (ASQ)
  - Bullied or cyber bullied (past 12 months)
  - Psychosis screen (past 12 months) – items from the Diagnostic Interview Schedule for Children (DISC-IV)
  - Disordered eating (past 12 months)
  - Sleep (usual hours per night)
    1. Youth risk behaviour (past-month alcohol use, lifetime illicit substance use)
    2. Education
    3. Family information
  - Country of birth
  - Education, income, employment, housing tenure
  - Family functioning: partner, family blending, support (McMaster Family Assessment Device (FAD))
  - Family connectedness (self-rated as always, mostly, sometimes, hardly ever/never)

Area-level variables:

- - Green space access and quality
  - Condition of the home
  - School Index of Community Socio-Educational Advantage (ICSEA)
  - Synthetic SA1-level small area estimate (SAE) of prevalence of internalising disorders e.g., major depression and anxiety disorders. See Appendix 4 for summary of small area estimation of internalising disorder prevalence (among Australians aged 12-17) at SA1-level.
  - Aggregated data from person-level data

**Auxiliary data (aggregated at SA1-level)**

ABS Census data (2011, 2016):

- - Country of birth
  - Aboriginal and/or Torres Strait Islander Status
  - Parent country of birth
  - Income
  - Employment
  - Education
  - Housing tenure (owned outright, owned with mortgage, rental – non-government, rental – state, other)

Socio-Economic Indexes for Areas (SEIFA) (ABS Census data; 2011, 2016):

- - Index of Relative Socio-economic Disadvantage (IRSD)
  - Index of Relative Socio-economic Advantage and Disadvantage (IRSAD)
  - Index of Economic Resources (IER)
  - Index of Education and Occupation (IEO)

ARIA remoteness index (major cities, inner regional, outer regional, remote, very remote). ARIA stands for Accessibility and Remoteness Index of Australia.

The quality of candidate variables for use in modelling was assessed by inspecting cross tabulations of variables by self-harm status and assessing the distribution (number (%)) of observations per cell and level of missingness. Where sparse distributions were observed, and categories were considered similar, some categories were collapsed to derive a version of the variable more suited for inclusion in modelling. Auxiliary variables were derived from six ABS domains. For each domain, proportions were derived from the count and total reported for that domain. The distributions of these variables were summarised overall (i.e., across all SA1s with data), and by self-harm status in the YMM dataset. Most had skewed distributions; summary statistics were presented as medians (interquartile range (IQR)) with means provided for comparison. Differences in distributions by self-harm status in YMM dataset were tested using Wilcoxon’s rank sum test.

For proportions with adequate distributions, categorical variables were derived based on rounded approximate quartiles of the distribution. For sex, areas with the highest quartile of “proportion male” were compared to all other areas (lower three quartiles). The cut-point that corresponded to this dichotomisation was 57%. Cut-points based on approximate quartiles were sometimes rounded to more interpretable cut-points e.g., 10% or 30%. Some categories of SA1-level variables were collapsed based on expert opinion and literature review. For example, the middle three quintiles of the SEIFA decile indices were collapsed to facilitate comparison with the highest and lowest quintile.

## Appendix 2. External Validation of Synthetic Self-Harm Prevalence Estimates

The synthetic self-harm prevalence estimates derived from the final model were aggregated to Statistical Area Level 2 (SA2-level) and compared to SA2-level average adult suicide death data from the National Mortality Database (NMD) (Australian Institute of Health and Welfare, nd) using linear regression.

Scatter plots of mean suicide rate (square-root transformed) by SA2 Small Area Estimates (SAEs) for self-harm aggregate irrespective of intent (2011; primary outcome) and suicide attempts (2019; secondary outcome) are shown in Figure S1 with fitted linear regression lines. All self-harm outcomes (primary and secondary) were significantly associated with mean crude suicide rates (*p*<.001), with regression slopes ranging from 1.5 (95% CI:1.0–2.0) for self-harm (irrespective of intent, primary outcome) in 2019 (Adjusted R^2^=0.015) to 2.7 (95% CI: 2.1–3.2) for suicidal ideation/plans in 2019 (Adjusted R^2^=0.044).


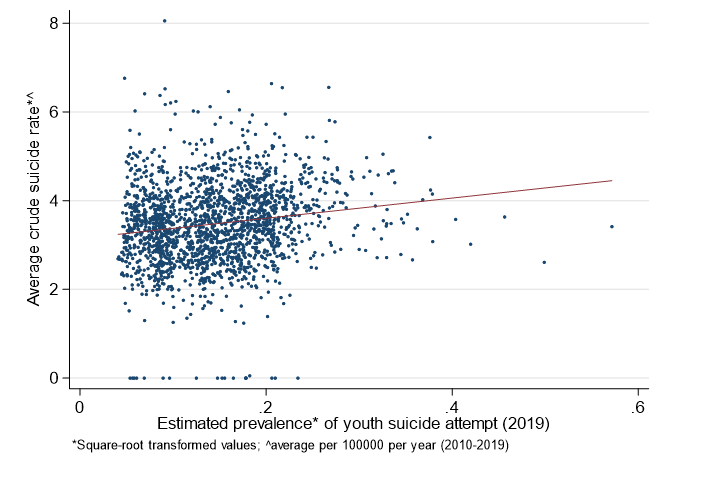


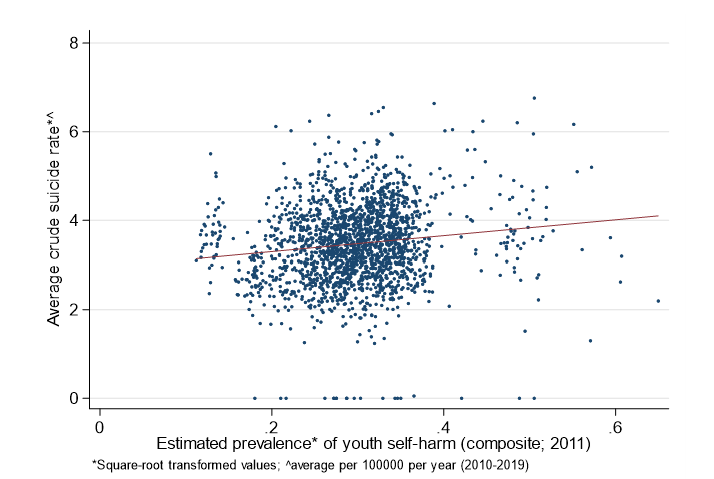


**Figure S1.** Scatterplots of mean suicide rates by youth suicide attempt (2019; top) and self-harm aggregate (irrespective of intent) outcome (2011; bottom) (square-root transformed) by SA2 self-harm small area estimates (SAEs) with fitted regression lines.

## Appendix 3. Additional Results Tables and Figures

**Table S1.** Estimated crude self-harm prevalence (% (95% CI)), by age and sex derived from the YMM nationwide survey

| **Age** | **Sex** | **Self-harm aggregate^a^** | **Non-suicidal Self-harm** | **Suicidality^b^** | **Ideation/Plans^c^** | **Attempts** |
| --- | --- | --- | --- | --- | --- | --- |
| 12-15 | Male | 3.6 (2.4-5.3) | 3.0 (1.9-4.6) | 3.4 (2.3-5.2) | 2.6 (1.7-4.1) | 0.8 (0.4-1.9) |
| 16-17 | Male | 7.8 (6.0-9.9) | 6.2 (4.7-8.1) | 6.8 (5.2-8.9) | 3.9 (2.8-5.6) | 2.9 (1.8-4.5) |
| 12-17 | Male | 4.9 (3.9-6.1) | 4.0 (3.1-5.1) | 4.5 (3.5-5.8) | 3.0 (2.3-4.1) | 1.5 (1.2-2.0) |
| 12-15 | Female | 9.9 (7.7-12.6) | 9.8 (7.6-12.5) | 8.1 (6.0-10.8) | 5.4 (3.7-7.6) | 2.7 (1.7-4.4) |
| 16-17 | Female | 17.7 (15.1-20.7) | 16.8 (14.1-19.8) | 15.4 (12.7-18.6) | 10.7 (8.3-13.6) | 4.7 (3.3-6.6) |
| 12-17 | Female | 12.7 (10.9-14.7) | 12.3 (10.5-14.3) | 10.7 (9.0-12.6) | 7.3 (5.9-9.0) | 3.4 (2.5-4.7) |

^a^ Self-harm aggregate: self-harm with or without suicidal intent (non-suicidal self-harm or attempts) in the past 12 months (primary outcome); ^b^ Suicidality: suicidal ideation, plans, or attempts in the past 12 months; ^c^ Ideation/plans: suicidal ideation or planning only in the past 12 months (suicide attempts excluded from variable); 95% CI = 95% confidence interval; YMM = Young Minds Matter.

**Table S2.** Univariable and multivariable associations between individual-level variables and individual-level self-harm (n=2,578)

| **Variable** | **Category** | **Univariable** | **p-value** | **Multivariable** | **p-value** |
| --- | --- | --- | --- | --- | --- |
|  |  | **OR (95% CI)** |  | **OR (95% CI)** |  |
| Sex | Male | Reference |  | Reference |  |
|  | Female | 2.9 (2.2-3.8) | <0.001 | 1.8 (1.2-2.6) | 0.002 |
| Self-report depression (12 months) ^a^ | No | Reference |  | Reference |  |
|  | Yes | 23.3 (16.7-32.6) | <0.001 | 3.9 (2.5-6.0) | <0.001 |
| Self-report psych distress (ABS cat) ^b^ | Low | Reference | <0.001* | Reference | <0.001* |
|  | Moderate | 2.3 (1.4-4.0) | 0.002 | 1.5 (0.9-2.7) | 0.151 |
|  | High | 15.0 (9.1-24.8) | <0.001 | 5.0 (2.8-8.7) | <0.001 |
|  | Very high | 62.5 (35.5-110.0) | <0.001 | 9.3 (4.8-18.1) | <0.001 |
| Parent-report depression (12 months) ^a^ | No | Reference |  | Reference |  |
|  | Yes | 7.8 (5.3-11.5) | <0.001 | 2.0 (1.3-3.1) | 0.001 |
| Illicit substance use (lifetime) | No | Reference | <0.001* | Reference | 0.002* |
|  | Yes | 5.6 (4.1-7.6) | <0.001 | 2.4 (1.5-4.1) | 0.001 |
|  | Missing | 0.6 (0.3-1.0) | 0.064 | 1.2 (0.6-2.4) | 0.536 |
| Drank alcohol (past month) | No | Reference |  | Reference |  |
|  | Yes | 3.4 (2.5-4.6) | <0.001 | 1.7 (1.1-2.8) | 0.028 |
| Bullied or cyber bullied (12 months) | No | Reference |  | Reference |  |
|  | Yes | 3.6 (2.7-4.8) | <0.001 | 1.5 (1.0-2.2) | 0.039 |
| Family connectedness ^c^ | Always | Reference | <0.001* | Reference | 0.001* |
|  | Mostly | 3.0 (2.1-4.5) | <0.001 | 1.5 (1.0-2.4) | 0.080 |
|  | Sometimes | 11.0 (7.2-16.8) | <0.001 | 2.3 (1.4-3.7) | 0.001 |
|  | Hardly ever/never | 24.0 (13.9-41.4) | <0.001 | 3.6 (1.6-7.7) | 0.001 |
| Building conditions in area ^d^ | Immaculate | Reference |  | Reference | 0.010* |
|  | Well-kept | 0.7 (0.5-1.0) | 0.056 | 0.5 (0.4-0.8) | 0.006 |
|  | Fair | 0.8 (0.5-1.2) | 0.220 | 0.6 (0.4-1.0) | 0.042 |
|  | Poor | 1.9 (0.9-4.1) | 0.114 | 1.6 (0.6-4.3) | 0.323 |
| Family category | Two biological parents | Reference | <0.001* | Reference | 0.078* |
|  | Blended/step/other | 2.4 (1.6-3.7) | <0.001 | 1.9 (1.1-3.2) | 0.015 |
|  | Sole parent | 1.8 (1.3-2.5) | <0.001 | 1.0 (0.7-1.6) | 0.824 |
| Aboriginal and/or Torres Strait Islander Status | - | 1.7 (1.0-2.9) | 0.068 | - | - |
| Remoteness ^e^ | - | 1.1 (0.8-1.5) | 0.59 | - | - |

Note. Aboriginal and/or Torres Strait Islander Status and Remoteness did not remain in the multivariable model; *Overall Wald p-value; YMM = Young Minds Matter Survey; OR = odds ratio; 95% CI = 95% confidence interval.

^a^ Diagnostic Interview Schedule for Children (DISC-IV) major depression (youth-reported and parent-reported, past 12 months); ^b^ Psychological distress ABS categorises (low, moderate, high, very high), as measured using Kessler-10-item psychological distress scale (K-10); ^c^ Family connectedness: self-rated by young people in the YMM survey (The next few questions are about your family: ‘Do you feel like there are people who are there for you?’ All the time/always, most of the time, sometimes, hardly ever, never); ^d^ Building conditions in area: YMM interviewer-rated based on buildings in the area (‘What is the general condition of most of the buildings in this SA1?’ Immaculate, well-kept & clean (in good repair), fair, poor and in need of repair, badly deteriorated); ^e^ ARIA remoteness index (major cities, inner regional, outer regional, remote).

**Table S3.** Distribution and univariate associations between auxiliary variables and individual-level self-harm in the YMM sample (n=2,578 individuals in 540 SA1s)

| **SA1-level Variable** | **Category** | **Proportion** | **95% CI** | **OR (95% CI)** | **p-value** |
| --- | --- | --- | --- | --- | --- |
| IRSD quintile ^a^ | Lowest quintile | 0.16 | 0.13-0.21 | Reference | 0.66* |
|  | Quintile 2 | 0.19 | 0.15-0.24 | 0.8 (0.5-1.4) | 0.46 |
|  | Quintile 3 | 0.18 | 0.14-0.23 | 0.7 (0.4-1.2) | 0.23 |
|  | Quintile 4 | 0.23 | 0.18-0.28 | 0.7 (0.4-1.1) | 0.16 |
| IRSAD quintile ^a^ | Highest quintile | 0.23 | 0.19-0.29 | 0.8 (0.5-1.3) | 0.34 |
|  | Lowest quintile | 0.16 | 0.12-0.20 | Reference | 0.51* |
|  | Quintile 2 | 0.18 | 0.15-0.23 | 0.7 (0.4-1.2) | 0.25 |
|  | Quintile 3 | 0.21 | 0.17-0.26 | 0.7 (0.4-1.1) | 0.15 |
|  | Quintile 4 | 0.22 | 0.17-0.27 | 0.7 (0.4-1.1) | 0.12 |
| IER quintile ^a^ | Highest quintile | 0.23 | 0.18-0.28 | 0.8 (0.5-1.3) | 0.38 |
|  | Lowest quintile | 0.13 | 0.10-0.16 | Reference | 0.19* |
|  | Quintile 2 | 0.15 | 0.12-0.19 | 0.9 (0.5-1.6) | 0.78 |
|  | Quintile 3 | 0.17 | 0.14-0.21 | 0.6 (0.3-1.1) | 0.08 |
|  | Quintile 4 | 0.25 | 0.21-0.30 | 0.7 (0.4-1.2) | 0.21 |
| IEO quintile ^a^ | Highest quintile | 0.30 | 0.25-0.35 | 0.9 (0.5-1.5) | 0.73 |
|  | Lowest quintile | 0.20 | 0.16-0.25 | Reference | 0.075* |
|  | Quintile 2 | 0.21 | 0.17-0.26 | 0.7 (0.5-1.1) | 0.13 |
|  | Quintile 3 | 0.22 | 0.17-0.27 | 0.6 (0.4-1.0) | 0.049 |
|  | Quintile 4 | 0.22 | 0.18-0.27 | 0.6 (0.4-0.9) | 0.008 |
| SAE prevalence - internalising  disorders ^b^ | Highest quintile | 0.14 | 0.11-0.19 | 0.6 (0.4-1.0) | 0.047 |
|  | Lowest quintile | 0.14 | 0.10-0.18 | Reference | 0.014* |
|  | Quintile 2 | 0.25 | 0.21-0.29 | 1.4 (0.9-2.3) | 0.13 |
|  | Quintile 3 | 0.21 | 0.17-0.25 | 1.4 (0.9-2.3) | 0.13 |
|  | Quintile 4 | 0.22 | 0.18-0.27 | 1.8 (1.1-3.0) | 0.01 |
|  | Highest quintile | 0.19 | 0.15-0.23 | 2.2 (1.4-3.5) | 0.001 |
| Remoteness | Major Cities | 0.63 | 0.59-0.68 | Reference | 0.97 |
|  | Inner Regional | 0.27 | 0.22-0.32 | 1.0 (0.7-1.4) | 0.87 |
|  | Outer Regional | 0.08 | 0.06-0.12 | 1.0 (0.5-2.1) | 1.0 |
|  | Remote | 0.02 | 0.01-0.03 | 1.2 (0.5-3.0) | 0.66 |
| % Aboriginal and/or Torres Strait Islander Status  Persons (12-17 years) | <1% | 0.68 | 0.63-0.73 | Reference | 0.003* |
|  | 1-<5% | 0.05 | 0.03-0.09 | 1.8 (1.0-3.1) | 0.035 |
|  | 5%-<10% | 0.13 | 0.10-0.17 | 1.8 (1.2-2.8) | 0.004 |
|  | ≥10% | 0.13 | 0.10-0.17 | 1.5 (1.0-2.1) | 0.027 |
| Low-income families ^c^ | <10% | 0.27 | 0.22-0.32 | Reference | 0.16* |
|  | 10-<30% | 0.50 | 0.68-0.78 | 0.7 (0.5-0.1) | 0.06 |
|  | ≥30% | 0.23 | 0.19-0.27 | 0.8 (0.5-0.2) | 0.24 |
| Parents born overseas  (either parent) | <30% | 0.68 | 0.63-0.73 | Reference |  |
|  | ≥30% | 0.32 | 0.27-0.37 | 0.7 (0.5-1.0) | 0.045 |
| Sex | <57% male | 0.72 | 0.67-0.76 | Reference |  |
|  | ≥57% male | 0.28 | 0.24-0.33 | 0.9 (0.6-1.2) | 0.46 |
| Single parent, not employed | <20% single parent not employed | 0.92 | 0.89-0.94 | Reference |  |
|  | ≥20% single parent, not employed | 0.08 | 0.06-0.11 | 1.8 (1.2-2.8) | 0.006 |
| Blended family ^d^ | <10% | 0.76 | 0.71-0.81 | Reference | 0.63 |
|  | 10-<20% | 0.20 | 0.17-0.25 | 1.1 (0.8-1.5) | 0.53 |
|  | ≥20% | 0.03 | 0.02-0.05 | 1.3 (0.6-2.9) | 0.43 |

Note. IRSD = Index of Relative Socio-economic Disadvantage; IRSAD = Index of Relative Socio-economic Advantage and Disadvantage; IER = Index of Economic Resources; IEO = Index of Education and Occupation; SAE = small area estimation; YMM = Young Minds Matter Survey; SA1 = Statistical Area Level 1; OR = odds ratio; 95% CI = 95% confidence interval; *Overall Wald p-value.

^a^ For all SEIFA indices (i.e., IRSD, IRSAD, IER, IEO), high scores indicate relatively low financial disadvantage. This includes IRSD (Index of Relative Socio-economic Disadvantage) where a high score indicates a relative lack of disadvantage, and IRSAD (Index of Relative Socio-economic Advantage/Disadvantage) where a high score indicates a relative lack of disadvantage and greater advantage overall; ^b^ Synthetic small area estimation (SAE) of internalising disorder prevalence, as based on a previous Young Minds Matter analysis (see Appendix 4). Internalising disorders are defined as those characterised by anxiety, depressive, and somatic symptoms (whereas externalising disorders are characterised primarily by actions in the external world, such as acting out, antisocial behaviour, disruptive conduct, hostility, and aggression); ^c^ According to the ABS, low family income is defined as annual household income <$52k; ^d^ According to the ABS, blended family is defined as a couple family containing two or more children, of whom at least one is the natural or adopted child of both members of the couple, and at least one is the step child of either partner in the couple.

## Appendix 4. Synthetic Internalising Disorder Estimates

**Summary of the methodology for developing synthetic estimates of prevalence of mental health disorders in children and adolescents in Australia**

Young Minds Matter (YMM survey, or the second Australian Child and Adolescent Survey of Mental Health and Wellbeing (YMM), provided detailed information on the prevalence and severity of mental disorders in children and adolescents aged 4-17 years. YMM surveyed over 6,000 families with children aged 4-17 years by visiting 550 communities across Australia. The survey results identified strong trends in prevalence by socio-economic characteristics of families and geographic areas. Mental health disorders were more common in families experiencing various types of social disadvantage, including in low-income families, families living in public housing, sole parent families, and families living in disadvantaged areas.

Although the survey visited a large and representative range of communities across the country, it was not possible to visit all communities. Estimates of the prevalence of mental disorders for individual communities can be very valuable for designing, locating and funding support services for children and adolescents experiencing mental health disorders and their families.

Synthetic estimates of the prevalence and severity of mental disorders have been produced by combining statistical modelling of factors associated with mental disorder status from the YMM data, with information on the geographic distribution of those factors from the 2011 and 2016 Census, and projecting forward using the most recent Estimated Residential Population figures provided by the ABS (for 30 June 2019).

Synthetic estimates have been calculated for internalising disorders (anxiety disorders and major depressive disorder), externalising disorders (Attention-deficit/hyperactivity disorder and conduct disorder) and all disorders at the following levels:

- disorder type by age group (4-11 years and 12-17 years) and sex
- disorder type by severity (mild, moderate, severe)
- disorder type by sex for 16-17 year-olds

Estimates have been calculated at the SA1 level, and then aggregated up to higher levels of geographic classification, including to SA2 and SA3 level, Primary Health Network (PHN) region level and Commonwealth Electoral Districts.

Due to restrictions on access to Census data, a mixed modelling approach was taken to producing the synthetic estimates, using a combination of individual level characteristics and characteristics of SA1s (small areas with average population around 250). Individual characteristics included in the model were age group, sex, Indigenous status and family type. Small area characteristics included in the model were SEIFA Index of Relative Socio-Economic Disadvantage (IRSD) quintiles, proportion of children and adolescents born overseas, proportion of children and adolescents with one or both parents born overseas, proportion of families by household income levels, education levels of parents, labour force status of parents and housing tenure.

The models were first fit using the YMM survey data, and were then applied to the 2016 Census data to estimate prevalence of disorders for every SA1 in Australia. These estimates were then projected forward to 2019 population counts based on the growth rate in number of children and adolescents living in each SA1 area.

While synthetic estimates are the best available small area data that can be used for service and program planning and delivery, the actual number of cases of disorder in any small area can vary from the model prediction if there are unique factors of that area that are not included in the model.

To facilitate using these estimates for service planning, estimates have been produced based on both the prevalence of disorders (the proportion of children and adolescents predicted to have a mental disorder irrespective of the size of the area) and the density of children and adolescents with disorders (number of prevalent cases per square kilometre) as illustrated in the example below for the Commonwealth Electoral District of Brand, in Western Australia.
